# Lung ultrasound for the diagnosis of community-acquired pneumonia in children

**DOI:** 10.1007/s00247-017-3910-1

**Published:** 2017-09-21

**Authors:** Jacob A. M. Stadler, Savvas Andronikou, Heather J. Zar

**Affiliations:** 1 Department of Paediatrics and Child Health, University of Cape Town, Cape Town, South Africa; 2 Department of Paediatric Radiology, Bristol Royal Hospital for Children, Upper Maudlin Street, Bristol, BS2 8BJ UK; 3 University of Bristol, Bristol, UK; 4 Department of Radiology, University of Cape Town, Cape Town, South Africa; 5 Department of Paediatrics and Child Health, Red Cross War Memorial Children’s Hospital, Cape Town, South Africa; 6 MRC Unit on Child and Adolescent Health, University of Cape Town, Cape Town, South Africa

**Keywords:** Children, Community-acquired pneumonia, Lung, Pneumonia, Ultrasound

## Abstract

Ultrasound (US) has been proposed as an alternative first-line imaging modality to diagnose community-acquired pneumonia in children. Lung US has the potential benefits over chest radiography of being radiation free, subject to fewer regulatory requirements, relatively lower cost and with immediate bedside availability of results. However, the uptake of lung US into clinical practice has been slow and it is not yet included in clinical guidelines for community-acquired pneumonia in children. The aim of this review is to give an overview of the equipment and techniques used to perform lung US in children with suspected pneumonia and the interpretation of relevant sonographic findings. We also summarise the current evidence of diagnostic accuracy and reliability of lung US compared to alternative imaging modalities in children and critically consider the strengths and limitations of lung US for use in children presenting with suspected community-acquired pneumonia.

## Introduction

Pneumonia remains the leading cause of death in children outside the neonatal period, but confirmation of a clinically suspected diagnosis, either to guide management or for consistent case definition in epidemiological and vaccine studies, remains problematic. Chest radiography is generally considered the first-line standard-of-care imaging modality to investigate suspected pneumonia, with alveolar consolidation or interstitial infiltrates combined with high serum C-reactive protein considered diagnostic for bacterial pneumonia. However, chest radiography cannot be considered a diagnostic gold standard due to wide inter- and intraobserver variability when interpreting results, differing radiologic manifestations of pneumonia and possible lack of sensitivity and specificity [1–7]. Due to the potentially harmful effects of radiation exposure, some clinical guidelines advise against the routine use of chest radiography in uncomplicated acute lower respiratory infections in childhood populations with high vaccination cover for *Haemophilus influenzae* type B and *Pneumococcus* [8, 9]. The use of chest radiography is further limited by the cost and expertise required for operating a radiology service.

Historically, ultrasound (US) has played a relatively minor role in pneumonia diagnosis, being viewed mostly as a complementary tool to standard radiography in complicated disease. More recently, decreased cost and increased availability of portable US technology as well as its potential to decrease radiation exposure has renewed interest in the use of lung US as a first-line imaging modality for the diagnosis of pneumonia, especially in children. Methods initially used in adult studies were adapted for use in children and feasibility, diagnostic accuracy and reliability have now been assessed in children in multiple settings. As the use of US is not subject to the same regulatory requirements as radiography and the cost of basic US technology is considerably lower than operating a basic radiology service, it has the potential to expand access to diagnostic imaging in low resource settings and could lead to overall cost savings. Clinician-driven use with immediate availability of results is another reason US may be favoured in certain settings. This article aims to describe the technique and sonographic findings used for US diagnosis of community-acquired pneumonia in children and summarise current evidence of its diagnostic accuracy and reliability.

## Technique and equipment

The type and size of the transducer depends on the age and size of the child. For an intercostal approach, small linear or micro-convex probes are preferred. In lung US, where the pleura and subpleural space are being assessed, a high-frequency transducer (5-15 MHz) is appropriate. Children can be scanned in the upright, supine or decubitus position. Scanning an uncooperative child can be challenging but is usually feasible. One approach is to scan the child while he is seated on a caregiver’s lap (even while breastfeeding) to minimise anxiety. To improve control of the probe, the base of the operator’s hand can be stabilized against the chest wall. This minimises movement of the probe in an uncooperative child and improves visualisation.

If US is used as a primary imaging modality, a structured systematic approach is recommended to ensure both lungs are visualized completely. This differs from a focused approach taken when assessing a specific region of suspected pathology identified on prior imaging. A number of authors have used an approach similar to the technique described by Copetti and Cattarossi [10], which divides each hemithorax into anterior, lateral and posterior zones, subdivided into upper and lower halves. Each zone is then scanned along anatomical lines: parasternal, mid-clavicular, anterior axillary, mid-axillary, posterior axillary, mid-scapular and paravertebral. The lung is visualised through the intercostal window and the probe is rotated both perpendicular and parallel to the ribs and moved from one intercostal space to the next, usually in a caudal direction from the apices to the costophrenic angles. If an area of pathology is visualised, a focused assessment of that area is done. Dependent lung areas, which change according to patient positioning, should specifically be checked to exclude a pleural effusion. When scanning the posterior chest, it is helpful to have the patient move their shoulders forward to expose as much of the retro-scapular regions as possible.

In our experience, anatomical orientation can be difficult during lung US as the operator usually sees only part of any structure at any given time, but knowing where the probe is placed on the patient helps the operator identify which structures are being visualized. A good approach is to start in the upper zones to ensure the probe is over lung and identify the pleural line deep to the ribs and then move the probe caudally until the subdiaphragmatic organs are seen. When the probe is held still at the lung base, the diaphragmatic line and abdominal structures can be seen moving in and out of view with respiration. This appearing and disappearing of aerated lung is referred to as the curtain sign. This is helpful in distinguishing the subdiaphragmatic viscera from lower lobe consolidation, a common pitfall when assessing the lower chest zones.

## Ultrasound findings in pneumonia

**Fig. 1:**
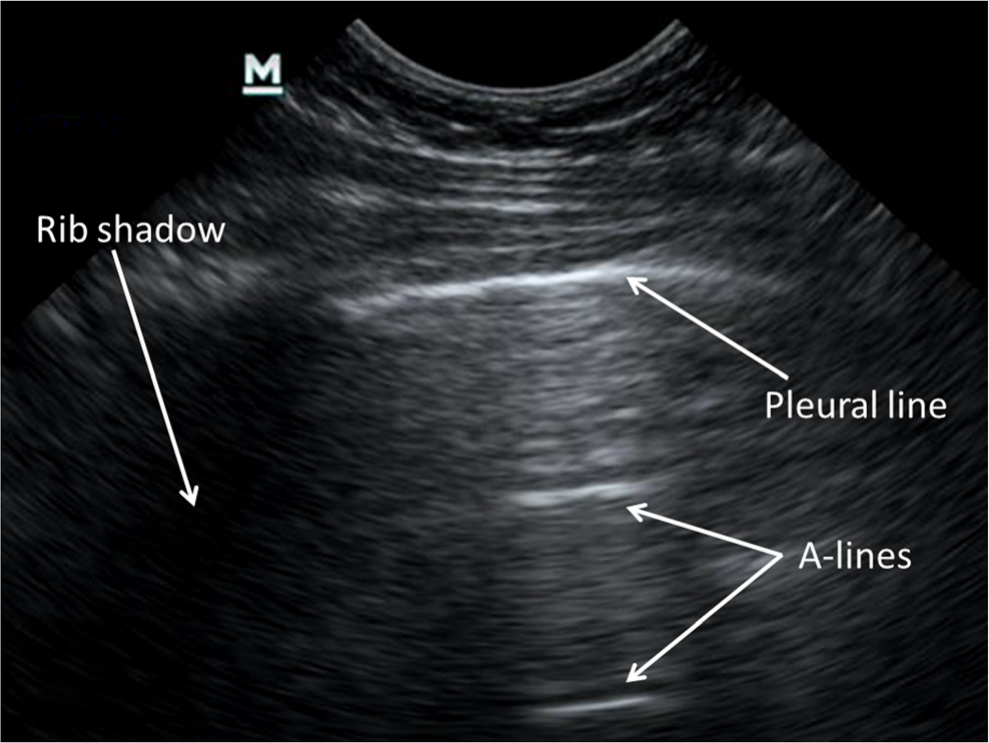
An ultrasound image from the right anterior upper lung zone in a 16-month-old girl demonstrates normal lung echo pattern with a smooth, hyperechoic pleural line, A-lines and no B-lines

**Fig. 2:**
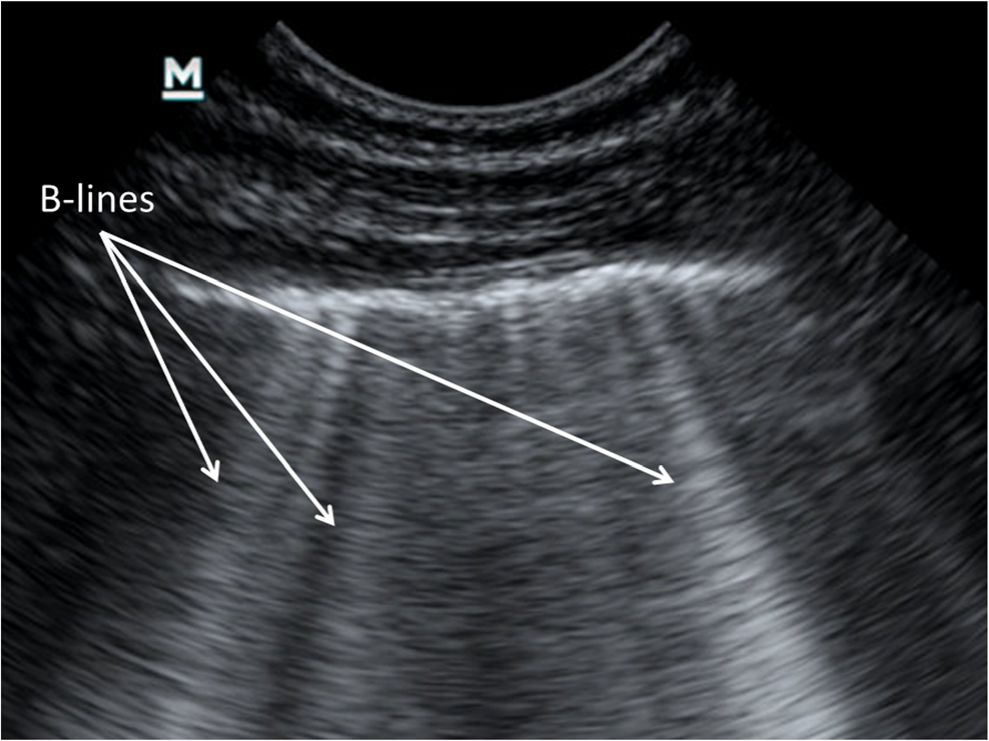
An ultrasound image from the left anterior lower lung zone in a 2-year-old boy who presented with symptoms of pneumonia shows multiple and confluent B-lines in a single view representing an interstitial disease pattern

In healthy aerated lung, only the pleura can be directly visualised by US, appearing as a smooth hyperechoic line deep to the ribs. The US beam cannot penetrate calcified bone and the ribs cast an acoustic shadow that is displayed as an anechoic segment deep to each rib. Visualisation of the visceral pleura sliding over the parietal layer during respiration, referred to as lung sliding, gives the pleural line a shimmering appearance. When lung sliding is absent, a pneumothorax should be suspected and can be confirmed with a number of measures, the detail of which falls outside the scope of this article. Normal air-filled lung parenchyma cannot be directly visualised by US, but gives rise to a characteristic artefactual pattern known as A-lines: hyperechoic lines running parallel to the pleural line that are, in fact, reverberation artefacts of the pleural line (Fig. 1). B-lines (alternatively referred to as lung comets or comet-tail artefacts) are hyperechoic lines arising from and running perpendicular to the pleura up to the deep edge of the image, without fading, and obliterating the A-lines where they cross. Lichtenstein [11] initially suggested that increased B-lines originate from thickened, oedematous interlobular septa. However, more recent experimental work suggests the origin of B-lines not to be from distinct anatomical structures, but rather from arbitrary air-fluid interfaces produced in the lung parenchyma by adjacent fluid and air-filled structures such as alveolar air and interstitium, which become increasingly dense with a corresponding increase in extravascular lung water or decrease in aeration [12–14]. On a macroscopic level, B-lines correlate with thickened interlobular septae or ground-glass appearance identified on computed tomography (CT) [11, 15]. Although occasional B-lines are seen in normal lung, especially in dependent zones, a distinct increase in the amount and density of B-lines is considered pathological. Three or more separate B-lines visualised at once (at the same time in any view) (Fig. 2) or when they become confluent (also referred to as compact B-lines) have been correlated with thickening of the interlobular septae due to increased interstitial fluid or infiltration using CT in adults [11, 16]. A generalised picture of pathological B-lines is referred to as interstitial syndrome, but pathological B-lines can also occur in localised regions depending on the underlying pathological process [11]. It must be remembered that increased B-lines are a nonspecific feature that cannot reliably distinguish underlying pathology, such as distinguishing exudative from transudative causes of interstitial oedema or an infective from a noninfective inflammatory process.

**Fig. 3:**
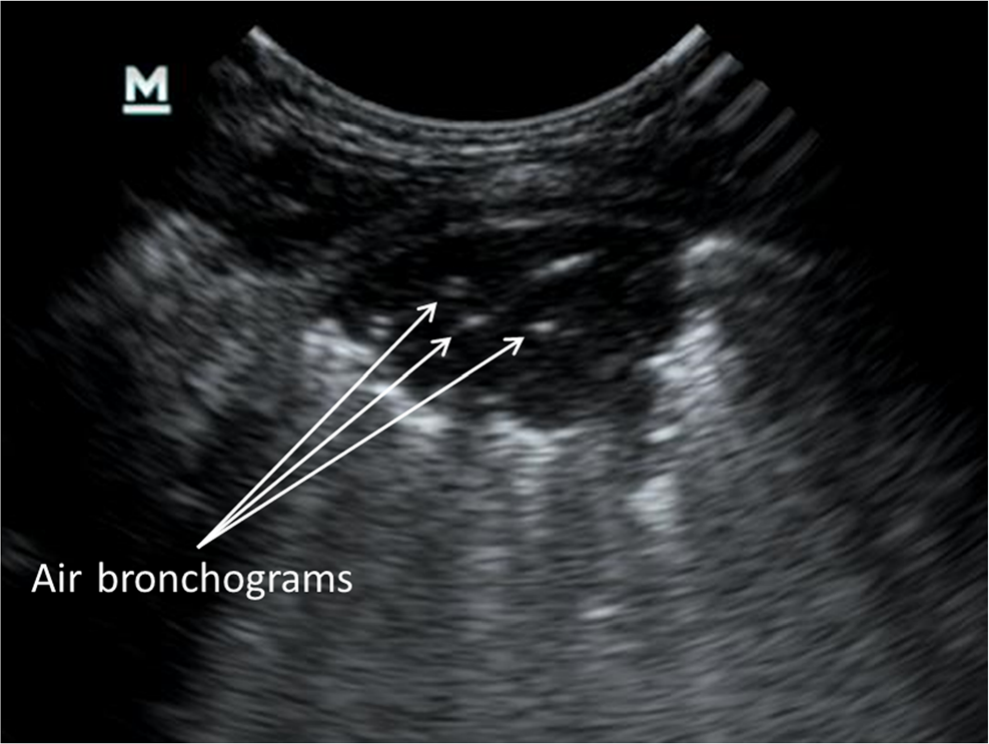
An ultrasound image from the right posterior upper lung zone in a 3-month-old girl hospitalised with pneumonia shows a wedge-shaped hypoechoic area of subpleural consolidation. Associated features that can be seen are air bronchograms represented by punctate hyperechoic specs within the lesion, a hypoechoic pleural line over the lesion and multiple B-lines that arise from the deep edge of the consolidation rather than from the pleura

Infective and inflammatory processes can cause fluid to displace alveolar air. When this process of air-space consolidation extends to the pleura, it can be visualised with US, usually as a poorly circumscribed, hypoechoic subpleural area with a number of associated features (Fig. 3). These include: a) loss of pleural line echogenicity over the area of consolidation and the absence of A-lines within the area, b) increased B-lines surrounding the area of consolidation, c) B-lines often arising from the deep edge of the consolidation rather than from the pleura and d) sonographic air bronchograms seen as multiple hyperechoic punctate or lenticular specs within the area of consolidation or branching tree-like structures depending on the plane at which they are cut by the US beam. Large consolidations tend to have a characteristic liver-like appearance, referred to as hepatisation. Atelectasis or lung collapse has a similar appearance to consolidation. A number of associated features have been described that can potentially distinguish pneumonic consolidation from collapse, but our experience and that of other authors is that the distinction cannot reliably be made, especially when small areas of consolidation are considered [17–19]. A pleural effusion presents as anechoic or hypoechoic fluid in the pleural space, with or without internal structures and debris. US is particularly sensitive for identifying even very small effusions and can be used to characterise effusions by demonstrating the presence of loculations and fibrin stranding [20].

Although both consolidation and interstitial syndrome have been described in various non-infective conditions in adults, in the context of a febrile child with respiratory symptoms, both these sonographic patterns are usually considered diagnostic of lower respiratory tract infection. In the studies evaluated for this review, consolidation, air bronchograms and pleural effusions were considered diagnostic of bacterial pneumonia by most authors. In both adult and paediatric studies, there appears to be general acceptance of the notion that an interstitial pattern represents viral disease [21, 22]. While this interpretation is consistent with the World Health Organization’s standardized interpretation of chest radiographs [5], direct microbiological evidence linking interstitial pattern to specific pathogens is lacking. Air bronchograms were not always required to be present to define a consolidation, but some authors consider consolidation without bronchograms to be atelectasis. When a distinction was drawn between consolidation and atelectasis, it was not always clear whether the latter was considered a diagnostic feature of pneumonia. Some authors also drew a distinction between consolidations smaller or bigger than 1 cm, raising a question about the diagnostic value of detecting very small areas of sonographic consolidation [21, 23, 24].

## Comparison with chest radiography and computed tomography

A number of studies have assessed the diagnostic performance of lung US compared to chest radiographs for the diagnosis of pneumonia, usually defined as the presence of air-space consolidation on either modality. A recent meta-analysis by Pereda et al. [25] showed overall pooled sensitivity of 96% (95% confidence interval [CI]: 94–97%) and specificity of 93% (95% CI: 90–96%), and positive and negative likelihood ratios of 15.3 (95% CI: 6.6–35.3) and 0.06 (95% CI: 0.03–0.11), respectively, when lung US was compared with a reference standard of either chest radiography alone or a combination of chest radiographs, clinical and laboratory findings [25]. The meta-analysis included eight studies with a combined total of 795 children and median age of 0.03–5.6 years. In subgroup analysis, both expert and novice clinician-sonographers achieved high rates of diagnostic accuracy with sensitivity and specificity above 90% in both groups. More recent studies that compared lung US and chest radiographs reported similar findings. A number of these studies did not report traditional accuracy measures, but reported agreement between chest radiographs and lung US instead, showing substantial agreement with kappa values of 0.64–0.89 [26, 27]. Several studies investigated and reported the reasons for discordant results. Positive lung US findings but negative chest radiograph findings occurred due to: 1) superior sensitivity of lung US to detect very small (subcentimetre) consolidations [21, 24, 26, 28]; 2) retro-cardiac or subdiaphragmatic consolidations not visible on posterior-anterior chest radiographs where no lateral views were available [29] and 3) misclassification of liver or spleen as lower lobe consolidation [21]. Negative lung US findings but positive chest radiograph findings occurred due to: 1) consolidations not reaching the pleural surface (often located in the perihilar or paracardiac regions) [24, 27] and 2) consolidations located in regions hard to reach with lung US such as retro-scapular, supraclavicular or axillary regions [30, 31].

The reliability of lung US in children was assessed by four studies, which reported moderate to substantial interobserver agreement for the interpretation of lung US images by multiple readers with kappa values of 0.55–0.93 [21, 23, 32, 33]. Only one study directly compared interobserver agreement of lung US with chest radiographs on the same set of patients, with kappa of 0.55 for lung US compared to kappa of 0.36 for chest radiographs for the detection of lung consolidation [33]. Similarly, fair to moderate interobserver agreement for the interpretation of chest radiographs for paediatric pneumonia with kappa values less than 0.6 is frequently reported in the literature, although varying considerably depending on level of prior training of reporters [1–4]. No study reported intraobserver agreement for lung US interpretation.

A common limitation in all these studies is the lack of a true diagnostic reference standard against which lung US and chest radiographs findings could be validated. Although CT would be an ideal gold standard, its routine use to diagnose paediatric pneumonia cannot be justified. However, three studies reported a small number of cases where chest CT had been performed on clinical indications [10, 28, 34]. In at least five of these cases, CT confirmed those US findings that were discordant with chest radiography. One recent study compared sensitivity and specificity of lung US and chest radiography for a number of pathological findings (including consolidation, interstitial disease and pleural effusion) against CT in 132 children ages 3 months to 18 years [33]. Only 36 patients actually had a CT done (based on clinical indications) and, of these, only 6 children had a primary clinical diagnosis of pneumonia while the majority (33) also had an underlying complex chronic condition, including malignancy (11) and chronic respiratory conditions (9). Latent class modelling was used to do a partial reference standard analysis to estimate sensitivity and specificity for the full sample. Sensitivity estimates were not found to be statistically significantly different between lung US and chest radiographs for detecting consolidation, interstitial disease or pleural effusion. However, in this study, chest radiographs had significantly better specificity estimates than lung US for excluding consolidation, interstitial disease and pleural effusion. Comparative studies using CT as reference standard have more frequently been performed in adults, and a recent meta-analysis published on the topic included a total of 1172 patients from 10 studies in which CT was used as reference standard, either in all cases or to validate discordant US and radiographic results [35]. This meta-analysis reported pooled sensitivity and specificity for lung US at 94% (95% CI interval: 92–96%) and 96% (95% CI: 94–97%), respectively, and pooled positive and negative likelihood ratios of 16.8 (95% CI: 7.7–37.0) and 0.07 (95% CI: 0.05–0.10), respectively. Although there are important differences to consider between adults and children with regard to pneumonia epidemiology and anatomy, it is not unreasonable to expect that similar results would be found in children if CT were routinely used as reference standard.

## Methodological considerations

The quality of all the studies included in the meta-analysis of Pereda et al. [25] was reported as high but with significant methodological heterogeneity. Compliance with the Standards for Reporting of Diagnostic Accuracy Studies (STARD) guidelines across studies included in this review was variable, at times lacking details of the definitions and rationale for diagnostic criteria for each imaging modality and the choice of reference standard, which made comparison of results across studies problematic. Differences in statistical analysis and reporting further complicated the comparison of results. Ideally, comparing performance characteristics of diagnostic tests is done by comparing both index tests against the same definitive reference standard, which then allows for a direct comparison of the accuracy measures calculated for each. A number of studies in this review used chest radiography as the reference standard [21, 26, 27, 36]. In this instance, the calculated accuracy of the index test (lung US) needs to be compared against the implied 100% accuracy of the reference standard (chest radiography). It is also important to note that accuracy calculated in this way will be limited by the underlying diagnostic accuracy of chest radiography (a relatively poor reference standard) as it assumes chest radiograph findings to be a true measure of the condition. In fact, calculating accuracy measures in this way would penalise lung US with a false-positive finding if it detects abnormality not evident on chest radiographs. One study, therefore, opted to exclude sonographic consolidations with diameter <1 cm from the analysis as these are considered undetectable by chest radiography [21]. Several authors stated that they did not consider this the most meaningful way of comparing accuracy due to the inherent diagnostic limitations of chest radiographs [10, 29]. Instead, positive and negative detection rates and agreement between modalities (raw agreement indices and/or kappa statistics) were reported [10, 27, 29, 31, 34]. In the absence of a strong objective reference standard (such as CT), some authors opted to compare both modalities to a composite final diagnosis of pneumonia based on expert opinion considering clinical presentation and course of disease as well as radiologic and laboratory findings [28, 37–39]. This allowed for direct comparison of accuracy measures between modalities. However, using chest radiograph findings as part of the reference standard introduces incorporation bias, which would inflate accuracy measures for chest radiographs. As lung US findings were not incorporated in a similar way when determining the final diagnosis, accuracy measures for lung US are not affected by this bias. A number of other commonly occurring biases also need to be considered. In all studies in this review, participants were selected exclusively among hospital inpatients (the emergency room, paediatric ward or intensive care unit) and, in most cases, chest radiographs were performed based on the clinical need for imaging. These two factors both self-select for the clinically more severe spectrum of disease (spectrum bias), which also tends to inflate accuracy measures of diagnostic tests. In a number of studies, the investigators who interpreted lung US results were not masked for clinical information. This would introduce reader bias. Some authors considered the use of only experienced clinicians/sonographers to perform and interpret scans, a threat to the generalisability of their results. However, studies that used clinicians with multiple levels of prior training and experience reported a high level of accuracy and substantial inter-rater reliability independent of prior experience.

Recent studies comparing lung US and chest radiographs are summarised in Table 1.

**Table 1:** Summary of recent studies investigating the use of lung ultrasound (LUS) for diagnosis of community-acquired pneumonia (CAP) in children

Author Year Country	*n* Gender (M:F) Mean age	Clinical setting	LUS definition for CAP	CXR definition for CAP	Final diagnosis of CAP (Reference standard for calculating sensitivity and specificity)	Main outcomes reported	Results	Main limitations
**Urbankowska et al.** [27] 2015 Poland	106 (39:67) 52.5 months	Hospital	Consolidation with or without bronchograms.	Consolidation; patchy infiltrates; interstitial infiltrates; pleural effusion.	CXR	Detection rates and agreement compared between CXR and LUS. Sensitivity and specificity. Positive predictive value and negative predictive value.	CXR+: 76/106 LUS+: 71/106 Overall agreement: kappa=0.89 Sensitivity: 93% Specificity: 100% Positive Predictive Value: 100% Negative Predictive Value: 85.7%	CXR used as reference standard. Possible selection bias - included only cases requiring referral to hospital. Inter-reader reliability not assessed.
**Ho et al.** [30] 2015 Taiwan	163 (91:72) 73.2 months	Hospital (paediatric ward and ICU)	Consolidation with bronchograms; pleural effusion.	Radiologist opinion (detail not specified).	Sensitivity and specificity not reported.	Detection rates and agreement compared between CXR and LUS.	CXR+: 152/163 (0.93) LUS+: 159/163 (0.98) Overall agreement: 147/163 (0.90)	Possible selection bias: included only hospitalised cases. Retrospective design with up to 48-h delay between CXR and LUS. Inter-reader reliability not assessed.
**Ianniello et al.** [31] 2016 Italy	84 (44:40) 72 months	Hospital ER	Consolidation and interstitial syndrome.	Air-space consolidation and interstitial disease pattern.	Sensitivity and specificity not reported.	Detection rates and agreement compared between CXR and LUS.	CXR+ rate: 47/84 LUS+ rate: 60/84 Overall agreement: 70/84	Relatively small sample size. Retrospective study design.
**Guerra et al.** [29] 2016 Italy	222 (108:114) 58.8 months	Hospital ER	Consolidation with air bronchograms.	Parenchymal consolidation or focal ground-glass opacity.	Sensitivity and specificity not reported.	Detection rates and agreement compared between CXR and LUS.	CXR+: 197/222 LUS+: 207/222 Proportion overall agreement: 0.89 Positive agreement: 0.94 Negative agreement: 0.40	Possible selection bias: included only cases referred to hospital and further excluded mild cases. Inter-reader reliability not assessed.
**Boursiani et al.** [37] 2017 Greece	69 (27:42) 54 months	Hospital ER	Alveolar or interstitial disease pattern.	Alveolar or interstitial disease pattern.	Expert opinion based on overall clinical picture.	Sensitivity, specificity and ROC curve.	CXR sensitivity: 96% CXR specificity: 100% LUS sensitivity: 92% LUS specificity: 100% ROC curve not significantly different between CXR & LUS.	Subjective reference standard. Possible selection bias: CXR part of reference standard. Relatively small sample size.
**Claes et al.** [24] 2017 Belgium	143 (77:66) 36 months	Hospital ER	Air-space consolidation.	Air-space consolidation	CXR (consolidation only).	Agreement, sensitivity, specificity, positive and negative predictive values and ROC curve.	Sensitivity: 98% Specificity: 92% Positive predictive value: 85% Negative predictive value: 99% ROC curve: AUC=0.95 Overall Agreement: 134/143	CXR used as reference standard. Possible selection bias: included only cases requiring referral to hospital and requiring CXR. Inter-reader reliability not assessed.

*CXR* chest radiography, *ER* emergency room, *F* female, *ICU* intensive care unit, *M* male, *ROC* receiver operating characteristic

## Strengths and limitations of lung ultrasound

Despite evidence of diagnostic accuracy and reliability compatible or better than chest radiography for detecting lung consolidation, the uptake of lung US into clinical practice has been slow and US is not yet included in clinical management guidelines for community-acquired pneumonia in children. Assessing the overall value of a diagnostic test is much more complex than merely assessing diagnostic accuracy and reliability. The overall value is often measured by assessing the impact on a range of clinical and nonclinical outcomes. Therefore, it is important to consider the strengths and limitations of lung US in addition to the diagnostic performance.

The immediate bedside availability of results provided by lung US is generally considered a strength, but it is offset by the time required to perform the scan, which counts as additional time clinicians have to spend per patient. A median time of 6.4–10 min per scan has been reported, with no significant difference between experienced and novice operators [21, 23, 24, 28]. A recent randomised controlled trial also demonstrated that although clinicians spent a bit more time per patient, the overall length of stay in the emergency department was shortened with the use of lung US (with optionally added chest radiography) [32]. This study also showed a 38% reduction in chest radiograph use with no statistically significant difference in the rates of unscheduled health care visits, missed pneumonia cases or adverse events (death or resuscitation required) between the interventional arm where lung US was performed first and chest radiographs optional and the control arm where chest radiographs and lung US were both performed routinely. The usefulness of lung US for following resolution of lung consolidation has also been demonstrated in a number of studies in children and presents a further opportunity for decreasing use of chest radiography [27, 30, 31].

A major feasibility concern is the learning curve and training requirements for clinicians to perform and interpret lung US in children. A number of authors said their training of clinicians involved theoretical training focused on disease recognition and potential pitfalls as well as supervised practical training [21, 23, 26, 32]. A recent study showed that US performed well in the hands of general practitioners after they received individualised training over a 7-day period from an expert radiologist [23]. Other studies suggest that focused training as short as 1 h is adequate to produce highly accurate results with no statistically significant difference between novice and experienced operators [21, 25]. Although it appears that the duration of training minimally affects results, it is our experience that adequate training should not be underestimated and that there is indeed a learning curve to confidently perform and interpret lung US scans. Supervised training and quality assurance by logging and reviewing scans with an experienced operator is therefore advised as part of any training program. Other limitations of lung US already discussed in this article include the inability to visualise consolidations not extending to the pleura or in areas covered by bony structures, the inability to reliably distinguish consolidation from atelectasis and the potential overdiagnosis of pneumonia due to the ability of lung US to detect very small subcentimetre consolidations of which the pathological relevance is uncertain. Also important is the inability of lung US to demonstrate certain features routinely assessed on chest radiographs of children presenting with respiratory distress including hyperinflation, cardiac size and shape as well as airway position, size and patency [19].

## Conclusion

Evidence suggests that when performed by adequately trained clinicians a structured lung US examination can detect lung consolidation and other features suggestive of pneumonia in children with the similar accuracy and reliability as chest radiographs, but with the added benefits of no exposure to ionizing radiation and potential savings in cost and time. In settings where chest radiography is not available, lung US may fill an important diagnostic gap for children presenting with suspected pneumonia. A small amount of evidence also suggests that it is indeed safe in children with suspected pneumonia to substitute chest radiography with lung US while still keeping the option of chest radiography open based on clinical judgement. In a setting where both modalities are available as diagnostic options, lung US holds the potential to decrease the use of chest radiographs, both during diagnosis and follow-up of children with pneumonia. However, there are a number of clinically relevant questions that the current literature does not fully address: for example, how to determine when a negative lung US requires further evaluation with chest radiographs or whether it is safe not to prescribe antibiotics in cases of suspected pneumonia when lung US is normal and only shows interstitial syndrome or very small sonographic consolidations. Evidence also clearly shows that lung US has inherent limitations that make it impossible for it to completely replace chest radiographs when investigating children with respiratory symptoms. It further emphasizes the importance of using clinical judgement to interpret imaging findings in context when using lung US to guide clinical management. Prospective studies of childhood pneumonia should consider validation of a diagnostic and management algorithm that integrates use of lung US and chest radiographs and aims to use US findings to guide antibiotic therapy. Consideration should also be given to developing a standardised interpretation method for lung US findings in childhood pneumonia, similar to the World Health Organization’s standardised interpretation of chest radiographs, for the purpose of ensuring consistent case definitions in studies investigating the role of lung US in the diagnosis and management of childhood pneumonia.

## References

[1] Bada C, Carreazo NY, Chalco JP (2007). Inter-observer agreement in interpreting chest X-rays on children with acute lower respiratory tract infections and concurrent wheezing. Sao Paulo Med J.

[2] Johnson J, Kline JA (2010). Intraobserver and interobserver agreement of the interpretation of pediatric chest radiographs. Emerg Radiol.

[3] Edwards M, Lawson Z, Morris S (2012). The presence of radiological features on chest radiographs: how well do clinicians agree?. Clin Radiol.

[4] Levinsky Y, Mimouni FB, Fisher D (2013). Chest radiography of acute paediatric lower respiratory infections: experience versus interobserver variation. Acta Paediatr.

[5] Cherian T, Mulholland EK, Carlin JB (2005). Standardized interpretation of paediatric chest radiographs for the diagnosis of pneumonia in epidemiological studies. Bull World Health Organ.

[6] Hagaman JT, Panos RJ, Rouan GW (2009). Admission chest radiograph lacks sensitivity in the diagnosis of community-acquired pneumonia. Am J Med Sci.

[7] Tanaka N, Emoto T, Suda H (2015). Community-acquired pneumonia: a correlative study between chest radiographic and HRCT findings. Jpn J Radiol.

[8] Harris M, Clark J, Coote N et al (2011) British Thoracic Society guidelines for the management of community acquired pneumonia in children: update 2011. Thorax 66(Suppl 2):ii1-ii23. doi:10.1136/thoraxjnl-2011-200598 10.1136/thoraxjnl-2011-200598 21903691

[9] National Collaborating Centre for Women’s and Children’s Health (UK) (2015) Bronchiolitis in children: diagnosis and management. National Institute for Health and Care Excellence, London pp 1–30 26065055

[10] Copetti R, Cattarossi L (2008). Ultrasound diagnosis of pneumonia in children. Radiol Med.

[11] Lichtenstein D, Meziere G, Biderman P (1997). The comet-tail artefact: an ultrasound sign of alveolar-interstitial syndrome. Am J Respir Crit Care Med.

[12] Soldati G, Giunta V, Sher S (2011). “Synthetic” comets: a new look at lung sonography. Ultrasound Med Biol.

[13] Soldati G, Inchingolo R, Smargiassi A (2012). Ex vivo lung sonography: morphologic-ultrasound relationship. Ultrasound Med Biol.

[14] Picano E, Pellikka PA (2016). Ultrasound of extravascular lung water: a new standard for pulmonary congestion. Eur Heart J.

[15] Martelius L, Heldt H, Lauerma K (2016). B-lines on pediatric lung sonography: comparison with computed tomography. J Ultrasound Med.

[16] Lichtenstein DA, Mezière GA (2008). Relevance of lung ultrasound in the diagnosis of acute respiratory failure the BLUE protocol. Chest.

[17] Riccabona M (2008). Ultrasound of the chest in children (mediastinum excluded). Eur Radiol.

[18] Toma P (2013). Lung ultrasound in bronchiolitis. Eur J Pediatr.

[19] Tomà P, Owens CM (2013). Chest ultrasound in children: critical appraisal. Pediatr Radiol.

[20] Calder A, Owens CM (2009). Imaging of parapneumonic pleural effusions and empyema in children. Pediatr Radiol.

[21] Shah VP, Tunik MG, Tsung JW (2013). Prospective evaluation of point-of-care ultrasonography for the diagnosis of pneumonia in children and young adults. JAMA Pediatr.

[22] Tsung JW, Kessler DO, Shah VP (2012). Prospective application of clinician-performed lung ultrasonography during the 2009 H1N1 influenza a pandemic: distinguishing viral from bacterial pneumonia. Crit Ultrasound J.

[23] Chavez MA, Naithani N, Gilman RH (2015). Agreement between the World Health Organization algorithm and lung consolidation identified using point-of-care ultrasound for the diagnosis of childhood pneumonia by general practitioners. Lung.

[24] Claes A-S, Clapuyt P, Menten R (2017). Performance of chest ultrasound in pediatric pneumonia. Eur J Radiol.

[25] Pereda MA, Chavez MA, Hooper-Miele CC (2015). Lung ultrasound for the diagnosis of pneumonia in children: a meta-analysis. Pediatrics.

[26] Esposito S, Papa SS, Borzani I (2014). Performance of lung ultrasonography in children with community-acquired pneumonia. Ital J Pediatr.

[27] Urbankowska E, Krenke K, Drobczyński Ł (2015). Lung ultrasound in the diagnosis and monitoring of community acquired pneumonia in children. Respir Med.

[28] Reali F, Sferrazza Papa GF, Carlucci P (2014). Can lung ultrasound replace chest radiography for the diagnosis of pneumonia in hospitalized children?. Respiration.

[29] Guerra M, Crichiutti G, Pecile P (2016). Ultrasound detection of pneumonia in febrile children with respiratory distress: a prospective study. Eur J Pediatr.

[30] Ho M-C, Ker C-R, Hsu J-H (2015). Usefulness of lung ultrasound in the diagnosis of community-acquired pneumonia in children. Pediatr Neonatol.

[31] Ianniello S, Piccolo CL, Buquicchio GL (2016). First-line diagnosis of paediatric pneumonia in emergency: lung ultrasound (LUS) in addition to chest-X-ray (CXR) and its role in follow-up. Br J Radiol.

[32] Jones BP, Tay ET, Elikashvili I (2016). Feasibility and safety of substituting lung ultrasonography for chest radiography when diagnosing pneumonia in children: a randomized controlled trial. Chest.

[33] Ambroggio L, Sucharew H, Rattan MS et al (2016) Lung ultrasonography: a viable alternative to chest radiography in children with suspected pneumonia? J Pediatr 176:93–98.e7. doi:10.1016/j.jpeds.2016.05.033 10.1016/j.jpeds.2016.05.033 27318374

[34] Seif El Dien HM, Abd ElLatif DAK (2013). The value of bedside lung ultrasonography in diagnosis of neonatal pneumonia. Egypt J Radiol Nucl Med.

[35] Chavez MA, Shams N, Ellington LE (2014). Lung ultrasound for the diagnosis of pneumonia in adults: a systematic review and meta-analysis. Respir Res.

[36] Iuri D, De Candia A, Bazzocchi M (2009). Evaluation of the lung in children with suspected pneumonia: usefulness of ultrasonography. Radiol Med.

[37] Boursiani C, Tsolia M, Koumanidou C (2017). Lung ultrasound as first-line examination for the diagnosis of community-acquired pneumonia in children. Pediatr Emerg Care.

[38] Liu J, Liu F, Liu Y (2014). Lung ultrasonography for the diagnosis of severe neonatal pneumonia. Chest.

[39] Caiulo VA, Gargani L, Caiulo S (2013). Lung ultrasound characteristics of community-acquired pneumonia in hospitalized children. Pediatr Pulmonol.

